# Substance use prevention among migrant children in Serbia- challenges and opportunities

**DOI:** 10.1093/eurpub/ckac131.516

**Published:** 2022-10-25

**Authors:** B Kilibarda, P Simonovic, A Medarevic, V Jovanovic, M Vasic, I Misic, J Zajeganovic-Jakovljevic

**Affiliations:** 1 Institute of Public Health of Serbia, Belgrade, Serbia; 2 UNICEF Serbia, Belgrade, Serbia

## Abstract

Among almost millions of children asylum seekers registered in the EU in 2015-2017, there were one-fifth unaccompanied children. These children are facing many challenges, including substance use while at the same time the recommendations for prevention are underdeveloped in many countries. To address the issue of substance use among migrant children in Serbia, the Institute of Public Health of Serbia with the support of the UNICEF and in cooperation with other partners developed recommendations for preventive activities in 2022 based on the migrant needs and country-specific situation. In the first phase of development, a desk review of current legislation and available relevant data was done. In the second phase, in order to gain in-depth view of the challenges and opportunities, workshops were conducted in two migrant centers with medical and other staff. Based on the findings and consultation process, feasible, evidence-based interventions were recommended. Several barriers to the implementation of evidence-based prevention activities in migrant centers were identified. To address the knowledge gap on the management of acute intoxication and the current referral system, guidelines were developed. The diversity of professional backgrounds of staff in migrant centers was identified as a barrier and recommendations for further training were made. There are several barriers that need further action and solutions such as short periods of stay in centers that hinder the provision of structured programs, language barriers, and ethical issues for minors, especially unaccompanied children. Prevention activities in migrant centers need to consider the specific needs of migrants, such as language barrier, capacities, and relevant knowledge of available staff and country-specific situations. The development of guidelines and a flowchart for a referral system for migrants at risk for substance abuse and dependence can be a useful tool for staff in migrant centers.

**Key messages:**

• Substance use prevention among migrants should be evidence-based and in line with migrant needs and country-specific situations.

• Guidelines for prevention should be available to migrant centers’ staff.

